# Single enantiomer synthesis of α-(trifluoromethyl)-β-lactam

**DOI:** 10.3762/bjoc.7.86

**Published:** 2011-06-06

**Authors:** Václav Jurčík, Alexandra M Z Slawin, David O'Hagan

**Affiliations:** 1 EASTChem School of Chemistry and Centre for Biomolecular Sciences, University of St Andrews, North Haugh, St Andrews, Fife, KY16 9ST, UK

**Keywords:** enantiomeric resolution, organofluorine building blocks, α-(trifluoromethyl)-β-lactam

## Abstract

The first synthesis of α-(trifluoromethyl)-β-lactam ((*S*)-**1**) is reported. The route starts from α-(trifluoromethyl)acrylic acid (**2**). Conjugate addition of α-(*p*-methoxyphenyl)ethylamine ((*S*)-**3b**), generated an addition adduct **4b** which was cyclised to β-lactam **5b**. Separation of the diastereoisomers by chromatography gave ((α*S,*3*S*)-**5b**). N-Debenzylation afforded the desired α-(trifluoromethyl)-β-lactam ((*S*)-**1**). The absolute stereochemistry of diastereoisomers **5** was determined by X-ray crystallographic determination of a close structural analogue, (α*S,*3*S*)-**5c**, and then ^1^H and ^19^F NMR correlation to the individual diastereoisomers of **5a** and **5b**.

## Introduction

**Figure 1:**
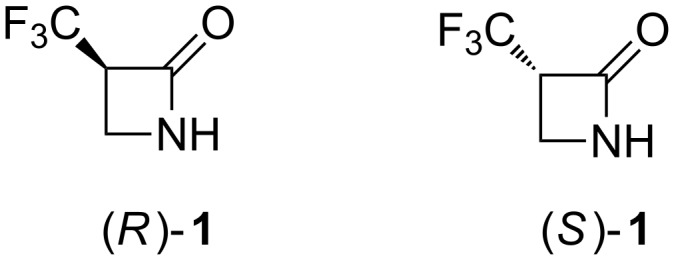
Enantiomers of α-(trifluoromethyl)-β-lactam (**1**).

β-Lactams (azetidin-2-ones) have played a prominent role in medicinal chemistry and many structural variants have been prepared and elaborated [1]. Similarly, the CF_3_ group is an ubiquitous substituent in pharmaceutical research, where it is used to modify the activity of a drug candidate or to block adventitious metabolism and improve the pharmacokinetic profiles [2–3]. Likewise, the substituent is found widely distributed in agrochemical products [4]. The majority of CF_3_ containing compounds reported in the literature are CF_3_-aryl, CF_3_-ether [5–6] or CF_3_-heteroaromatic in nature, and these substituents have contributed significantly to the fine chemicals and related industries. However, there is an increasing awareness and demand for molecular building blocks which carry the CF_3_ group at a stereogenic centre [7–8] and some such motifs are emerging in new chemical entities licenced for the pharmaceuticals market, but also in organic materials area, e.g., liquid crystals [9]. Methodologies continue to emerge for the asymmetric introduction of the CF_3_ group [10–13]. In this contribution, we report a synthesis of α-(trifluoromethyl)-β-lactam (**1**) which allows access to its enantiomerically pure forms, (*R*)-**1** and (*S*)-**1** as illustrated in Figure 1.

## Results and Discussion

**Scheme 1:**
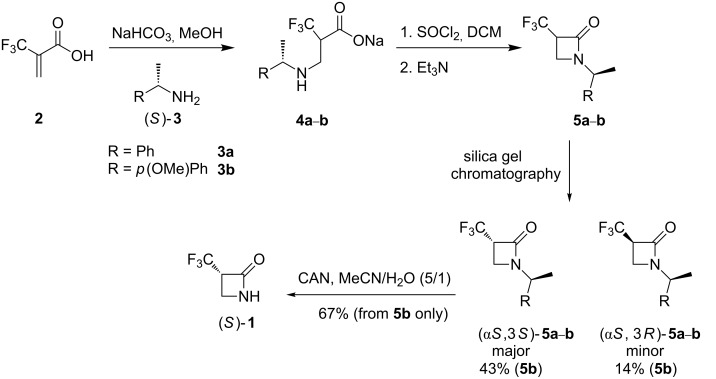
Synthetic route involving a diastereoisomeric separation to α-(trifluoromethyl)-β-lactam ((*S*)-**1**) from α-(trifluoromethyl)acrylic acid **2** and (*S*)-α-(*p*-methoxyphenyl)ethylamine (**3b**). Yields are quoted for the conversion of **4b**.

**Figure 2:**
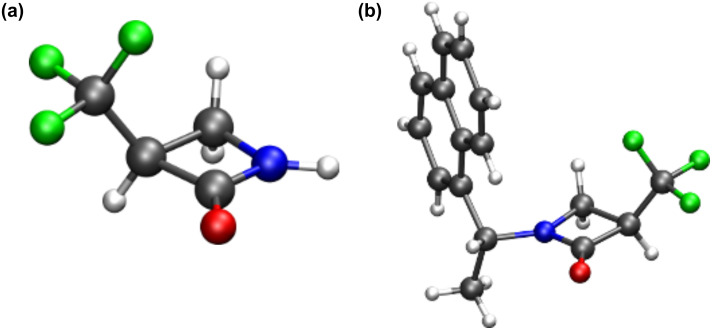
X-ray structures of (a) β-lactam (*S*)-**1** and (b) (α*R*,3*R*)-**5c**. (a) Determination of the absolute stereochemistry of (α*R*,3*R*)-**5c** by X-ray crystallography allowed an absolute assignment of β-lactam **1**.

The synthesis shown in Scheme 1 follows a strategy developed [14] for enantiomers of the methyl analogue of **1**, and starts from α-(trifluoromethyl)acrylic acid (**2**) which offered a commercially available source of the CF_3_ group. It was envisaged that conjugate addition of an enantiomerically pure amide such as (*R*)- or (*S*)-**3** would generate the addition adducts, carboxylate salts **4**, as a mixture of diastereoisomers. Cyclisation to the N-substituted β-lactams **5** would give a mixture of two stereoisomers which might be separated into their individual diastereoisomers **5** by chromatography. *N*-Benzyl deprotection of the individual diastereoisomers would then provide β-lactam **1** as a single enantiomer. At the outset, (*S*)-(α-phenyl)ethylamine (**3a**) was explored as the enantiomerically pure amine. In the event **4a** was generated after aza-Michael addition as a mixture of two stereoisomers, without any obvious diastereoisomeric bias (1:1, 0% de) as judged by both ^1^H and ^19^F NMR. β-Lactam ring closure, using thionyl chloride and triethylamine gave the *N*-methylbenzyl-β-lactam **5a** with a modest diastereoisomeric bias (~50% de) indicating some epimerisation of the α-trifluoromethyl stereogenic centre, to a thermodynamic product ratio. This was supported in an analytical reaction by the observation of deuterium exchange at this stereogenic centre after the addition of D_2_O to the reaction on work up. The major and minor diastereoisomers of **5a** were separated by careful chromatography on silica gel into single stereoisomers. Completion of the syntheses required hydrogenolysis of the (*S*)-*N*-methylbenzyl moiety of **5a**. A range of conditions and catalysts were explored for the hydrogenolysis of **5a**, however cleavage of the C–N bond proved very difficult and a satisfactory method could not be found. Therefore, (*S*)-α-(*p*-methoxyphenyl)ethylamine (**3b**) was explored as an alternative amine for the aza-Michael reaction, as removal of this amine using ceric ammonium nitrate (CAN) oxidation offered a milder deprotection method [15]. The aza-Michael reaction proved straightforward to generate **4b** and then cyclisation again using thionyl chloride and triethylamine gave β-lactams **5b** in a 40% de, presumably again a thermodynamically biased isomer ratio. The diastereoisomers of **5b** could be separated by careful chromatography over silica gel and this led to the recovery of each isomer as a major and a minor product. Finally, oxidative scission of the major β-lactam stereoisomer (α*S*,3*R*)-**5b**, using CAN was relatively straightforward generating β-lactam (*S*)-**1** in 67% yield. This novel β-lactam is a crystalline solid, and a suitable crystal was subjected to X-ray structure analysis. The structure of **1** is shown in Figure 2a.

Accordingly, (*S*)-α-(*p*-methoxyphenyl)ethylamine (**3b**) emerged as the more satisfactory amine over **3a** for the preparation of **1**, due to the straightforward benzylic cleavage. Enantiomeric purity analysis of the resultant β-lactam **1** was evaluated by ^19^F NMR using a europium chiral shift reagent [**Eu**(hfc)_3_]. The comparison of a racemic sample of **1** and then a sample after diastereoisomer separation of **5b** as described above, indicated that β-lactam **1** was prepared in an enantiomerically pure form. There was no evidence that benzylic cleavage of the (*S*)-α-(*p*-methoxyphenyl)ethyl moiety with CAN resulted in epimerisation at the stereogenic centre of the β-lactam.

**Scheme 2:**
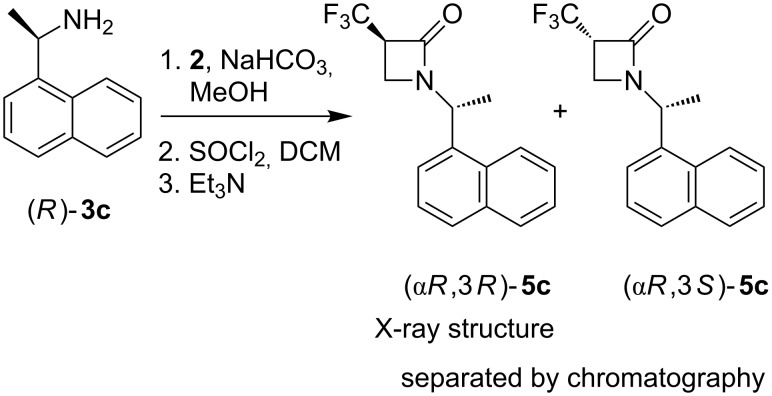
Synthesis of stereoisomers **5c**. The stereochemistry of the major isomer (α*R*,3*R*)-**5c** was solved by X-ray structure analysis.

Finally, it was necessary to determine the absolute configuration of the resultant β-lactam. To this end, X-ray crystallography of the major diastereoisomers of **5a** and **5b** was attempted after chromatographic separation. Despite considerable effort however we could not obtain crystals of single isomers of **5a** or **5b** suitable for X-ray structure analysis. Thus, a preparation of **5c** was carried out as illustrated in Scheme 2. The presence of the naphthyl ring in amine (*R*)-**3c** rendered the resultant β-lactam diastereoisomers **5c** more crystalline, and a single crystal X-ray structure was solved for the major-diastereomer revealing the (α*R*,3*R*)-**5c** configuration. The structure is shown in Figure 2b.

With this structural information in hand it was necessary to correlate to the stereoisomers of **5b**. The ^1^H and ^19^F NMR spectra of the major and minor diastereoisomers of **5a**–**c** were now compared. Clear chemical shift trends are observed as illustrated and tabulated in Table 1 and Table 2. For example, in all cases, the ^1^H NMR chemical shifts of the C-3 hydrogens (H_a_) of the β-lactam are shifted downfield in the major relative to the minor diastereoisomers. The non-equivalent faces of the planar β-lactam ring differentiates the two diastereotopic hydrogens at C-4 (H_b_ and H_c_).

**Table 1:** Comparison of the ^1^H NMR data of Hb and Hc of the diastereoisomers of **5a**–**c**.

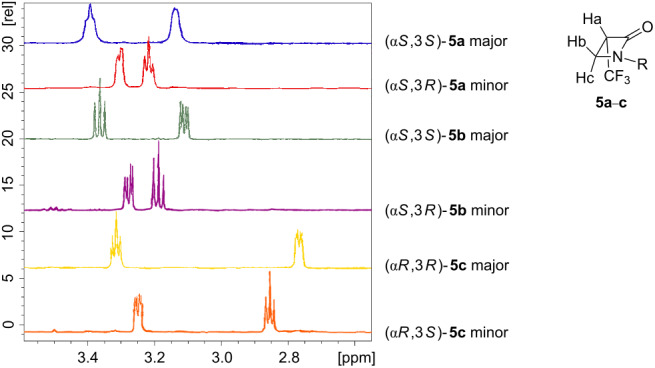							

	H_a_	H_b_			H_c_		
	δ ^1^H [ppm]	δ ^1^H [ppm]	*J*_1_ [Hz]	*J*_2_ [Hz]	δ ^1^H [ppm]	*J*_1_ [Hz]	*J*_2_ [Hz]

(α*S*,3*S*)-**5a** major	3.78	3.39	8.38	5.40	3.13	4.20	
(α*S*,3*R*)-**5a** minor	3.64	3.20	6.24	2.60	3.21	6.76	6.06
(α*S*,3*S*)-**5b** major	3.78	3.36	6.43	6.18	3.11	6.18	2.83
(α*S*,3*R*)-**5b** minor	3.73	3.27	6.47	2.45	3.18	6.55	5.27
(α*S*,3*R*)-**5c** major	3.79	3.31	7.49	6.61	2.77	6.40	2.48
(α*S*,3*S*)-**5c** minor	3.76	3.25	6.66	2.12	2.85	6.85	6.00

**Table 2:** Comparison of the ^19^F NMR chemical shifts of the diastereoisomers of **5a**–**c**.

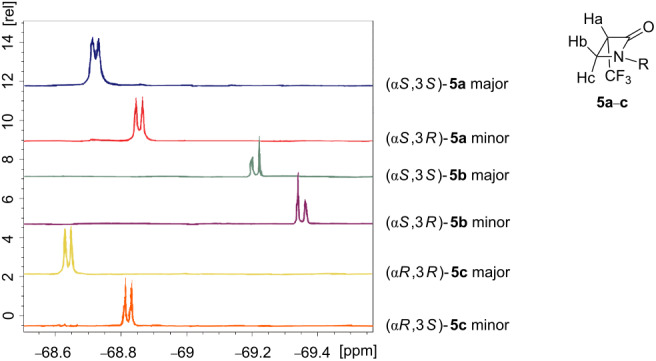				

	δ ^19^F **_major_** [ppm]	*J* [Hz]	δ ^19^F **_minor_** [ppm]	*J* [Hz]

**5a**	−68.72	8.73	−68.85	8.94
**5b**	−69.21	8.36	−69.35	8.36
**5c**	−68.62	9.14	−69.82	8.94

It is clear that for all three major isomers the ^1^H NMR signals for H_b_ and H_c_ have a larger chemical shift difference than that between the H_b_ and H_c_ signals of the minor isomers. Also the chemical shifts for H_b_ and H_c_ of the minor isomers lie within those of the major isomers. For the ^19^F NMR spectra shown in Table 2 the major isomers all have their trifluoromethyl signals downfield of the minor diastereoisomers.

On this basis, the NMR spectra of (α*R*,3*R*)-**5c**, where the absolute stereochemistry was determined by X-ray crystallography, allows the absolute stereochemistry of diastereoisomers **5a** and **5b** to be deduced by correlation, and it follows that benzylic cleavage of (*S*)-α-(*p*-methoxyphenyl)ethylamine (**3b**) gave rise to β-lactam (*S*)-**1**. Ready access to the (*R*)-**1** enantiomer would require starting the synthetic route from (*S*)-α-(*p*-methoxyphenyl)ethylamine (**3b**).

## Conclusion

In conclusion, a short single enantiomer synthesis of β-lactam (*S*)-**1** is reported which took advantage of an aza-Michael addition between (*S*)-α-(*p*-methoxyphenyl)ethylamine (**3b**) and α-(trifluoromethyl)acrylic acid (**2**). Cyclisation and then chromatographic resolution of the β-lactam diastereoisomers **5b**, followed by deprotection with ceric ammonium nitrate generated the β-lactam (*S*)-**1**. This contributes diversity to the known β-lactam motifs and incorporates the CF_3_ group.

## Experimental

### General

All reagents were obtained from commercial sources and were used without further purification unless otherwise stated. Air- and moisture-sensitive reactions were carried out under a positive pressure of argon in flame-dried glassware using standard Schlenk-line techniques. Dry CH_2_Cl_2_ was obtained from the Solvent Purification System MB SPS-800. Room temperature (RT) refers to 20–25 °C. Reaction temperatures of 0 °C were obtained in an ice/water bath. Reaction reflux conditions were obtained using an oil bath equipped with a contact thermometer. Solvent evaporations were carried out under reduced pressure on a Büchi rotary evaporator. Thin layer chromatography (TLC) was performed using Macherey-Nagel Polygram Sil G/UV254 plastic plates. Visualisation was achieved by inspection under UV light (255 nm). Column chromatography was performed using silica gel 60 (40–63 micron). NMR spectra were recorded on Bruker AVANCE 300, 400 or 500 MHz instruments. ^1^H and ^13^C NMR spectra were recorded in CDCl_3_ as solvent. ^19^F NMR spectra were referenced to CFCl_3_ as the external standard. Chemical shifts are reported in parts per million (ppm) and coupling constants (*J*) are given in Hertz (Hz). IR spectra were recorded on a Nicolet Avatar 360 FT-IR from a thin film (either neat or combined with nujol) supported between NaCl plates. Optical rotations [α]_D_ are given in 10^–1^ deg cm^2^ g^–1^ and were measured using a Perkin Elmer Model 341 polarimeter. Mass spectrometric (*m*/*z*) data was acquired by electrospray ionisation (ESI). High resolution mass analyses were recorded on a Micromass LCT TOF mass spectrometer using ES ionisation in positive ion mode.

#### General aza-Michael procedure

NaHCO_3_ (840 mg, 10.0 mmol) was added to a solution of α-(trifluoromethyl)acrylic acid (**2**, 1.41 g, 10.0 mmol) in methanol (10 mL), and then after 30 min stirring, a single enantiomer amine **3** (10.0 mmol) was added. The reaction was stirred at RT for 24 h. The solvent was evaporated under reduced pressure to afford the aza-Michael product as a colourless oil (quant) which solidified upon standing. Optionally, the sodium salt was converted to the amino acid HCl salt by treatment with aq HCl, followed by evaporation of the water and redissolving the residual in DCM.

#### Synthesis of aza-Michael adduct diastereoisomers 4a

General procedure with α-(trifluoromethyl)acrylic acid **2** (431 mg, 3.08 mmol), NaHCO_3_ (258 mg, 3.02 mmol) and (*S*)-phenylethylamine (381 μL, 3.02 mmol) in methanol (20 mL), gave after 96 h 830 mg (95%) of the title compound as a mixture of diastereoisomers (93% purity). Product **4a** was used without further purification. ^1^H NMR (400 MHz, CD_3_OD) 7.42–7.25 (m, 5H, Ar), 4.02–3.90 (m, 1H, PhC*H*CH_3_), 3.30–3.13 (m, 1H, CH_2_C*H*CF_3_), 3.10–2.91 (m, 1H, NC*H*_2_CH), 2.90–3.76 (m, 1H, NC*H*_2_CH), 1.44 (d, *J* = 6.7 Hz, 3H, C*H*CH_3_); ^13^C NMR (100 MHz, CD_3_OD) 172.3 (q, *J* = 2.0 Hz, *C*OONa), 172.2 (q, *J* = 2.0 Hz, *C*OONa), 143.9 (C-Ar), 143.5 (C-Ar), 129.9 (C-Ar), 129.8 (C-Ar), 128.9 (C-Ar), 128.8 (C-Ar), 128.1 (C-Ar), 128.0 (C-Ar), 126.8 (q, *J* = 278 Hz, *C*CF_3_), 126.9 (q, *J* = 278 Hz, *C*CF_3_), 59.6 (Ph*C*HCH_3_), 59.0 (Ph*C*HCH_3_), 52.6 (q, *J* = 25.0 Hz, *C*HCF_3_), 52.6 (q, *J* = 25.0 Hz, *C*HCF_3_), 52.1 (q, *J* = 25.0 Hz, *C*HCF_3_), 45.12 (q, *J* = 3.5 Hz, N*C*H_2_CH), 44.7 (q, *J* = 3.5 Hz, N*C*H_2_CH), 23.1 (CH*C*H_3_), 22.9 (CH*C*H_3_); ^19^F NMR (376 MHz, CD_3_OD) −69.0 (d, *J* = 9.3 Hz), −69.1 (d, *J* = 9.3 Hz); HRMS–ESI (*m*/*z*): Calcd for C_12_H_14_NO_2_F_3_Na, 284.0874; found, 284.0869.

#### Synthesis of aza-Michael adduct diastereoisomers 4b

General procedure with α-(trifluoromethyl)acrylic acid **2** (1.52 g, 10.8 mmol), NaHCO_3_ (912 mg, 10.8 mmol) and (*S*)-*p*-methoxyphenylethylamine (1.595 mL, 10.08 mmol) in methanol (20 mL), gave after 20 h 3.06 g (98%) of the title compound as a mixture of diastereoisomers (93% purity). The material was used without further purification. ^1^H NMR (400 MHz, CD_3_OD) 7.17–7.12 (m, 2H, Ar), 6.85–6.80 (m, 2H, Ar), 4.86 (q, *J* = 6.5 Hz, 1H, PhC*H*CH_3_), 3.74 (s, 3H, PhOC*H*_3_), 3.73–3.65 (m, 1H, CH_2_C*H*CF_3_), 3.27 (dd, *J* = 6.3 Hz, *J* = 6.3 Hz, 1H, NC*H*_2_CH), 3.01 (dd, *J* = 6.4 Hz, *J* = 2.7 Hz, 1H, NC*H*_2_CH), 1.52 (d, *J* = 6.7 Hz, 3H, C*H*CH_3_); ^13^C NMR (100 MHz, MeOD) 167.4 (N*C*OCH), 167.3 (N*C*OCH), 162.2 (C-Ar), 131.6 (C-Ar), 130.4 (C-Ar), 128.5 (C-Ar), 128.4 (C-Ar), 125.1 (q, *J* = 275 Hz, *C*F_3_), 125.5 and 125.1 (q, *J* = 275 Hz, *C*F_3_), 115.83 (C-Ar), 115.51 (C-Ar), 60.61 (Ph*C*HCH_3_), 60.55 (Ph*C*HCH_3_), 55.9 (PhO*C*H_3_), 48.2 (q, *J* = 30.2 Hz, *C*HCF_3_), 48.1 (q, *J* = 30.2 Hz, *C*HCF_3_), 42.6 (N*C*H_2_CH), 19.9 (CH*C*H_3_), 19.5 (CH*C*H_3_); ^19^F NMR (376 MHz, MeOD) −68.8 (d, *J* = 8.7 Hz, CHC*F*_3_), −68.9 (d, *J* = 8.1 Hz, CHC*F*_3_); HRMS–ESI (*m*/*z*): Calcd for C_13_H_16_NO_3_F_3_Na, 314.0980; found, 314.0975.

#### Synthesis of aza-Michael adduct diastereoisomers 4c

General procedure with α-(trifluoromethyl)acrylic acid **2** (236 mg, 1.69 mmol), NaHCO_3_ (142 mg, 1.685 mmol) and (*R*)-1-naphthylethylamine (295.4 mg, 1.685 mmol) in methanol (10 mL), gave after 96 h 550 mg (98%) of the title compound as a mixture of diastereoisomers (89% purity). The material was used without further purification. ^1^H NMR (400 MHz, CD_3_OD) 8.20 (t, *J* = 8.2 Hz, 1H, H-Ar), 7.91–7.86 (m, 1H, Ar), 7.83–7.79 (m, 1H, Ar), 7.72–7.68 (m, 1H, Ar), 7.59–7.46 (m, 3H, Ar), 4.99–4.88 (m, 1H, PhC*H*CH_3_), 3.35–3.22 (m, 1H, CH_2_C*H*CF_3_), 3.21–3.09 (m, 1H, NC*H*_2_CH), 3.03–2.94 (m, 1H, NC*H*_2_CH), 1.57 and 1.56 (d, *J* = 6.7 Hz, 3H); ^13^C NMR (100 MHz, CD_3_OD) 172.2 (*C*OONa), 172.0 (*C*OONa), 139.5 (C-Ar), 138.9 (C-Ar), 135.5 (C-Ar), 132.48 (C-Ar), 132.45 (C-Ar), 130.08 (C-Ar), 135.05 (C-Ar), 129.2 (C-Ar), 129.15 (C-Ar), 127.5 (C-Ar), 127.4 (C-Ar), 126.8 (C-Ar), 126.75 (C-Ar), 126.7 (C-Ar), 126.6 (C-Ar), 124.2 (C-Ar), 123.7 (C-Ar), 123.5 (C-Ar), 54.5 (Ph*C*HCH_3_), 54.1 (Ph*C*HCH_3_), 52.5 (q, *J* = 25.0 Hz, *C*HCF_3_), 52.2 (q, *J* = 25.0 Hz, *C*HCF_3_), 45.2 (q, *J* = 3.0 Hz, N*C*H_2_CH), 44.9 (q, *J* = 3.0 Hz, N*C*H_2_CH), 22.51 (CH*C*H_3_), 22.46 (CH*C*H_3_); ^19^F NMR (375 MHz, CD_3_OD) −69.0 (d, *J* = 9.7 Hz, CHC*F*_3_), −69.1 (d, *J* = 9.7 Hz, CHC*F*_3_); HRMS–ESI (*m*/*z*): Calcd for C_16_H_16_NO_2_F_3_Na, 334.1031; found, 334.1031.

#### General method for cyclisation of adducts 4 to β-lactams 5

The sodium salt of **4** (8.00 mmol) was dissolved in dry DCM (100 mL) and DMF (5 drops) was added. The mixture was cooled to 0 °C and then SOCl_2_ (2.78 mL, 40.00 mmol, 5 equiv) added dropwise. The reaction mixture was warmed to RT and stirred overnight. Solvent and excess of SOCl_2_ were removed under reduced pressure and the oily residue was redissolved in dry DCM (10 mL). The solution was heated to reflux while Et_3_N (5.38 mL, 39.75 mmol, 5 equiv) was added. The reaction mixture was diluted with water (10 mL) and extracted into DCM (3 × 10 mL). The combined organic phases were washed with brine (10 mL), dried (Na_2_SO_4_) and solvent was removed under reduced pressure to give **4** as a mixture of two isomers, which were then separated by chromatography.

#### Preparation of diastereoisomers 5a

Sodium salt **4a** (245 mg, 0.86 mmol) and SOCl_2_ (310 μL, 4.3 mmol) in DCM (20 mL), followed by Et_3_N (600 μL, 4.3 mmol), gave **5** as a yellow oily solid.

*Major isomer* (α*S*,3*S*)-**5a** (91 mg, 43%): [α]_D_^20^ −88.5 (*c* 0.34, CH_2_Cl_2_); IR (neat): 2978 (s), 1753 (vs), 1180 (s), 1120 (s), 892 (s), 699 (s), 571 (s) cm^−1^; ^1^H NMR (500 MHz, CDCl_3_) 7.42–7.27 (m, 5H, Ar), 4.97 (q, *J* = 7.0 Hz, 1H, ArC*H*CH_3_), 3.85–3.75 (m, 1H, C*H*CF_3_), 3.39 (dd, *J* = 7.3 Hz, *J* = 5.8 Hz, 1H, NC*H*_2_CH), 3.16–3.11 (m, 1H, NC*H*_2_CH), 1.64 (d, *J* = 7.0 Hz, 3H, CHC*H*_3_); ^13^C NMR (125 MHz, CDCl_3_) 159.4 (q, *J* = 4.0 Hz), 139.3 (C-Ar), 128.9 (C-Ar), 128.0 (C-Ar), 126.6 (C-Ar), 123.9 (q, *J* = 275 Hz, *C*F_3_), 52.0 (Ph*C*HCH_3_), 51.1 (q, *J* = 30.1 Hz, *C*HCF_3_), 37.6 (q, *J* = 3.1 Hz, N*C*H_2_CH), 18.2 (CH*C*H_3_); ^19^F NMR (470 MHz, CDCl_3_) −68.72 (d, *J* = 8.8 Hz, CHC*F*_3_); HRMS–ESI (*m*/*z*): Calcd for C_12_H_12_NOF_3_Na, 266.0769; found, 266.0769.

*Minor isomer* (α*S*,3*R*)-**5a** (29 mg, 14%): [α]_D_^20^ −36.7 (*c* 0.09, CH_2_Cl_2_); IR (neat): 2976 (s), 1756 (vs), 1184 (s), 1119 (s), 700 (s) cm^−1^; ^1^H NMR (500 MHz, CDCl_3_) 7.42–7.27 (m, 5H, Ar), 4.94 (q, *J* = 7.0 Hz, 1H, ArC*H*CH_3_), 3.80–3.71 (m, 1H, C*H*CF_3_), 3.30 (dd, *J* = 6.5 Hz, *J* = 2.2 Hz, 1H, NC*H*_2_CH), 3.22 (dd, *J* = 7.2 Hz, *J* = 6.0 Hz, 1H, NC*H*_2_CH), 1.67 (d, *J* = 7.1 Hz, 3H, CHC*H*_3_); ^13^C NMR (125 MHz, CDCl_3_) 159.3 (q, *J* = 4.2 Hz), 139.3 (C-Ar), 129.0 (C-Ar), 128.1 (C-Ar), 126.7 (C-Ar), 123.8 (q, *J* = 275 Hz, *C*F_3_), 52.5 (Ph*C*HCH_3_), 51.3 (q, *J* = 30.8 Hz, *C*HCF_3_), 37.7 (q, *J* = 3.1 Hz, N*C*H_2_CH), 18.4 (CH*C*H_3_); ^19^F NMR (470 MHz, CDCl_3_) −68.85 (d, *J* = 9.1 Hz, CHC*F*_3_); HRMS–ESI (*m*/*z*): Calcd for C_12_H_12_NOF_3_Na, 266.0769; found, 266.0766.

#### Preparation of diastereoisomers 5b

Sodium salt **4b** (1.18 g, 3.77 mmol) and SOCl_2_ (1.36 mL, 18.8 mmol) in DCM (100 mL) and DMF (5 drops), followed by Et_3_N (2.6 mL, 39.8 mmol), gave **5b** as a colourless oil.

*Major isomer* (α*S*,3*S*)-**5b** (447 mg, 43%): [α]_D_^20^ +96.5 (*c* 0.31, CH_2_Cl_2_); IR (neat): 2980 (s), 1756 (vs), 1612 (s), 1515 (s), 1372 (s), 1180 (s), 1120 (s), 1031 (s), 1010 (s), 835 (s) cm^−1^; ^1^H NMR (400 MHz, CDCl_3_) 7.30–7.25 (m, 2H, Ar), 6.95–6.90 (m, 2H, Ar), 4.94 (q, *J* = 6.5 Hz, 1H, PhC*H*CH_3_), 3.85 (s, 3H, PhOC*H*_3_), 3.84–3.73 (m, 1H, CH_2_C*H*CF_3_), 3.36 (dd, *J* = 6.0 Hz, *J* = 6.0 Hz, 1H, NC*H*_2_CH), 3.11 (dd, *J* = 6.3 Hz, *J* = 2.6 Hz, 1H, NC*H*_2_CH), 1.61 (d, *J* = 7.1 Hz, 3H, C*H*CH_3_); ^13^C NMR (100 MHz, CDCl_3_) 159.7 (N*C*OCH), 131.6 (C-Ar), 128.3 (C-Ar), 124.4 (q, *J* = 275 Hz, *C*F_3_) 114.6 (C-Ar), 55.8 (Ph*C*HCH_3_), 51.7 (PhO*C*H_3_), 51.4 (q, *J* = 30.2 Hz, *C*HCF_3_), 37.9 (q, *J* = 3.3 Hz, N*C*H_2_CH), 18.6 (CH*C*H_3_); ^19^F NMR (376 MHz, CDCl_3_) −69.2 (d, *J* = 8.1 Hz, CHC*F*_3_); HRMS–ESI (*m*/*z*): Calcd for C_13_H_14_NO_2_F_3_Na, 296.0874; found, 296.0868.

*Minor isomer* (α*S*,3*R*)-**5b** (140 mg, 14%): [α]_D_^20^ −42.1 (*c* 0.18, CH_2_Cl_2_); IR (neat): 2975 (s), 1760 (vs), 1611 (s), 1515 (s), 1370 (s), 1244 (vs), 1121 (s), 1031 (s), 1010 (s), 833 (s) cm^−1^; ^1^H NMR (400 MHz, CDCl_3_) 7.25–7.22 (m, 2 H, Ar), 6.94–6.90 (m, 2H, Ar), 4.91 (q, *J* = 7.0 Hz, 1H, PhC*H*CH_3_), 3.83 (s, 3H, PhOC*H*_3_), 3.78–3.68 (m, 1H, CH_2_C*H*CF_3_), 3.27 (dd, *J* = 6.4 Hz, *J* = 2.7 Hz, 1H, NC*H*_2_CH), 3.19 (dd, *J* = 6.3 Hz, *J* = 6.0 Hz, 1H, NC*H*_2_CH), 1.64 (d, *J* = 7.0 Hz, 3H); ^13^C NMR (100 MHz, CDCl_3_) 159.3 (N*C*OCH), 131.3 (C-Ar), 127.9 (C-Ar), 123.9 (q, *J* = 275 Hz, *C*F_3_), 114.2 (C-Ar), 55.3 (Ph*C*HCH_3_), 51.9 (PhO*C*H_3_), 51.0 (q, *J* = 31.2 Hz, *C*HCF_3_), 37.5 (q, *J* = 3.2 Hz, N*C*H_2_CH), 18.5 (CH*C*H_3_); ^19^F NMR (376 MHz, CDCl_3_) −69.35 (d, *J* = 8.1 Hz, CHC*F*_3_); HRMS–ESI (*m*/*z*): Calcd for C_13_H_14_NO_2_F_3_Na, 296.0874; found, 296.0883.

#### Preparation of diastereoisomers 5c

From sodium salt **4c** (500 mg, 1.50 mmol), SOCl_2_ (544 μL, 7.5 mmol) in DCM (20 mL), followed by Et_3_N (1.05 mL, 7.5 mmol) gave **5c** as a yellow solid.

*Major isomer* (α*R*,3*R*)-**5c** (192 mg, 41%): [α]_D_^20^ −40.9 (*c* 0.15, CH_2_Cl_2_); IR (neat): 2981 (s), 1760 (vs), 1368 (s), 1260 (s), 1158 (s), 1122 (s), 1011 (s), 780 (s) cm^−1^; ^1^H NMR (500 MHz, CDCl_3_) 8.10 (d, *J* = 8.4 Hz, 1H, Ar), 7.92 (d, *J* = 7.8 Hz, 1H, Ar), 7.88 (d, *J* = 8.4 Hz, 1H), 7.64–7.58 (m, 1H, Ar), 7.58–7.46 (m, 3H, Ar), 5.78 (q, *J* = 6.7 Hz, 1H, ArC*H*CH_3_), 3.82–3.73 (m, 1H, C*H*CF_3_), 3.31 (dd, *J* = 7.1 Hz, *J* = 6.0 Hz, 1H), 2.77 (dd, *J* = 6.5 Hz, *J* = 2.6 Hz, 1H), 1.80 (d, *J* = 7.2 Hz, 3H, CHC*H*_3_); ^13^C NMR (75 MHz, CDCl_3_) 159.3 (q, *J* = 4.3 Hz, N*C*OCH), 133.9 (C-Ar), 133.7 (C-Ar), 130.9 (C-Ar), 129.2 (C-Ar), 128.9 (C-Ar), 127.0 (C-Ar), 126.2 (C-Ar), 123.7 (q, *J* = 275 Hz, *C*F_3_), 123.7 (C-Ar), 122.7 (C-Ar), 51.0 (q, *J* = 31.1 Hz, *C*HCF_3_), 47.9 (Ph*C*HCH_3_), 37.4 (q, *J* = 3.2 Hz, N*C*H_2_CH), 17.8 (CH*C*H_3_); ^19^F NMR (470 MHz, CDCl_3_) −68.6 (d, *J* = 9.1 Hz, CHC*F*_3_); HRMS–ESI (*m*/*z*): Calcd for C_16_H_14_NOF_3_Na, 316.0925; found, 316.0915.

*Minor isomer* (α*R*,3*S*)-**5c** (70 mg, 15%): [α]_D_^20^ −35.7 (*c* 0.14, CH_2_Cl_2_); IR (neat): 2978 (s), 1755 (vs), 1366 (s), 1191 (s), 1120 (s), 778 (s) cm^−1^; ^1^H NMR (500 MHz, CDCl_3_) 8.15 (d, *J* = 8.6 Hz, 1H, Ar), 7.92 (d, *J* = 8.6 Hz, 1H, Ar), 7.87 (d, *J* = 8.5 Hz, 1H), 7.63–7.58 (m, 1H, Ar), 7.58–7.48 (m, 3H, Ar), 5.79 (q, *J* = 6.8 Hz, 1H, ArC*H*CH_3_), 3.69–3.60 (m, 1H, C*H*CF_3_), 3.25 (dd, *J* = 6.5 Hz, *J* = 2.5 Hz, 1H), 2.86 (dd, *J* = 6.9 Hz, *J* = 6.2 Hz, 1H), 1.82 (d, *J* = 7.2 Hz, 3H, CHC*H*_3_); ^13^C NMR (125 MHz, CDCl_3_) 159.2 (q, *J* = 4.5 Hz, N*C*OCH), 133.9 (C-Ar), 133.8 (C-Ar), 130.9 (C-Ar), 129.2 (C-Ar), 129.0 (C-Ar), 127.1 (C-Ar), 126.2 (C-Ar), 125.1 (C-Ar), 123.9 (q, *J* = 275 Hz, *C*F_3_), 123.7 (C-Ar), 122.6 (C-Ar), 47.7 (Ph*C*HCH_3_), 50.9 (q, *J* = 31.1 Hz, *C*HCF_3_), 37.2 (q, *J* = 3.0 Hz, N*C*H_2_CH), 17.7 (CH*C*H_3_); ^19^F NMR (470 MHz, CDCl_3_) −68.8 (d, *J* = 9.1 Hz, CHC*F*_3_); HRMS–ESI (*m*/*z*): Calcd for C_16_H_14_NOF_3_Na, 316.0925; found, 316.0919.

#### (*S*)-3-(Trifluoromethyl)azetidin-2-one (*S*)-1

To a solution of (α*S*,3*S*)-**5b** (325 mg, 1.18 mmol) in a mixture of MeCN (8 mL) and water (2 mL), ceric ammonium nitrate (1.94 g, 3.54 mmol, 3 equiv) was added portionwise. The reaction mixture was stirred at RT for 16 h and then quenched with sat. sodium bicarbonate (5 mL). After 10 min of stirring (when gas evolution ceased), the mixture was extracted into Et_2_O (3 × 10 mL), the combined organic phases were dried (Na_2_SO_4_) and the solvent was removed. Purification on silica gel (20% EtOAc in petrol) gave (*S*)-**1** as a colourless oil which solidified upon standing (110 mg, 67%). Mp 85 °C; [α]_D_^20^ +26.9 (*c* 0.09, CH_2_Cl_2_); IR (neat): 1707 (s), 1652 (s), 1180 (s), 1120 (s) cm^−1^; MS–ESI (*m*/*z*): 161.98 (M + Na); ^1^H NMR (400 MHz, CDCl_3_) 6.73 (bs, 1H, CH_2_N*H*CO), 3.91–3.81 (m, 1H, C*H*CF_3_), 3.47 (dd, *J* = 6.9 Hz, *J* = 6.3 Hz, 1H, NC*H*_2_CH), 3.37 (dd, *J* = 6.5 Hz, *J* = 3.0 Hz, 1H, NC*H*_2_CH); ^13^C NMR (100 MHz, CDCl_3_) 161.3 (q, *J* = 4.5 Hz, N*C*OCH), 123.7 (q, *J* = 275.8 Hz, *C*F_3_), 53.7 (q, *J* = 31.3 Hz, *C*HCF_3_), 36.7 (q, *J* = 3.5 Hz, N*C*H_2_CH); ^19^F NMR (376 MHz, CDCl_3_) −69.38 (d, *J* = 9.5 Hz, CHC*F*_3_); HRMS–ESI (*m*/*z*): Calcd for C_4_H_4_NOF_3_Na, 162.0143; found, 162.0147.

#### Crystallographic data

**1** C_4_H_4_F_3_NO, *M* = 139.08, Monoclinic, space group *P2(1)*, *a* = 9.226(11), *b* = 5.507(6), *c* = 11.229(12) Å, β = 99.401(13)°. *V* = 562.9(11) Å^3^, F(000) = 280, *Z* = 4, *D*_c_ = 1.641 Mg m^−3^, μ = 0.181 mm^−1^ (Mo-Kα, λ = 0.71073 Å). The data were collected at *T* = 93 (2) K, 5405 reflections (2.24 to 25.39°) were measured on a Rigaku Saturn 92 detector with 007 generator yielding 2016 unique data (*R*_int_ = 0.0777). Conventional *R* = 0.0194 for 1860 reflections with *I* ≥ 2σ, GOF = 1,011; 171 refined parameters. The largest peak in the residual map is 0.247 eÅ^−3^*.* Crystallographic data has been deposited with the Cambridge Crystallographic Data Centre as supplementary publication (CCDC 821176).

(α*R*,3*R*)-**5c** C_16_H_14_F_3_NO, *M* = 293.28, Monoclinic, space group *P2(1)*, *a* = 9.677(5), *b* = 6.094(3), *c* = 12.231(5) Å, β = 92.685(13)°. *V* = 720.5(5) Å^3^, F(000) = 304, *Z* = 2, *D*_c_ = 1.352 Mg m^−3^, μ = 0.949 mm^−1^ (Cu-Kα, λ = 1.54178 Å). The data were collected at *T* = 173 (2) K, 9149 independent reflections (3.62 to 25.35°) were measured on a Rigaku Saturn 92 detector with 007 generator yielding 2504 unique data (*R*_int_ = 0.0450). Conventional *R* = 0.0194 for 2416 reflections with *I* ≥ 2σ, GOF = 0.973; 191 refined parameters. The largest peak in the residual map is 0.124 eÅ^−3^*.* Flack parameter 0.08 (13). Crystallographic data has been deposited with the Cambridge Crystallographic Data Centre as supplementary publication (CCDC 821177).
